# 

**Published:** 1879-02

**Authors:** 


					﻿||	THE BUFFALO
MEDICAL AND SURGICAL
JOURNAL.
VOL. XVIII.
FEBRUARY, 1879.
NO. 7.
				

